# Tertiary lymphoid structures as biomarkers and therapeutic targets in neoadjuvant cancer immunotherapy

**DOI:** 10.3389/fimmu.2026.1882195

**Published:** 2026-07-01

**Authors:** Tong Wang, Wenlong Xu, Xiaofeng Wu

**Affiliations:** Department of Reproduction, Qingdao Hospital, University of Health and Rehabilitation Sciences (Qingdao Municipal Hospital), Qingdao, Shandong, China

**Keywords:** immune checkpoint blockade, neoadjuvant immunotherapy, predictive biomarker, tertiary lymphoid structures, tumor microenvironment remodeling

## Abstract

Neoadjuvant immunotherapy is reshaping the treatment of resectable solid tumors, but biomarkers that reflect spatially organized antitumor immunity remain limited. Tertiary lymphoid structures (TLS) are ectopic lymphoid aggregates that support local antigen presentation, lymphocyte recruitment, T–B cell interaction, germinal center reactions and humoral immunity. Increasing evidence suggests that TLS density, maturation, localization and cellular composition are associated with immune checkpoint blockade response, pathological regression and prognosis. Beyond their biomarker value, TLS may represent modifiable immune niches that can be induced or remodeled by checkpoint blockade, chemotherapy, radiotherapy, vaccines and stromal–vascular strategies. This mini-review discusses the biological basis, biomarker potential, therapeutic targeting and clinical translation of TLS in neoadjuvant cancer immunotherapy, highlighting key challenges and future TLS-guided trial designs.

## 1 Introduction

Neoadjuvant immunotherapy is reshaping the treatment paradigm of resectable solid tumors (1). Unlike adjuvant therapy, which is delivered after tumor removal, neoadjuvant treatment is administered while the primary tumor and its immune microenvironment remain intact (2). This setting may preserve a broader source of tumor antigens for immune priming, enable systemic antitumor immune activation before surgery, and provide resected specimens for direct pathological and translational assessment. Clinically, this strategy has shown meaningful activity across several tumor types (3, 4). In resectable non-small cell lung cancer, neoadjuvant nivolumab plus chemotherapy significantly improved event-free survival and pathological complete response compared with chemotherapy alone (5). In resectable stage III melanoma, neoadjuvant ipilimumab plus nivolumab improved event-free survival compared with standard adjuvant nivolumab (6). These studies support the concept that the preoperative window is not only a period for tumor reduction, but also an opportunity to remodel antitumor immunity.

However, reliable patient selection for neoadjuvant immunotherapy remains challenging. Current biomarkers, including PD-L1 expression, tumor mutational burden, CD8^+^ T-cell infiltration, interferon-related gene signatures and circulating tumor DNA, provide valuable but incomplete information (7). PD-L1 expression is spatially and temporally heterogeneous; tumor mutational burden does not necessarily reflect effective antigen presentation; CD8^+^ T-cell density alone cannot determine whether lymphocytes are functionally activated or spatially organized; and circulating tumor DNA mainly reflects tumor burden or molecular residual disease rather than local immune architecture (8). Therefore, biomarkers that integrate immune composition, spatial organization and functional state within the tumor microenvironment are urgently needed.

Tertiary lymphoid structures (TLS) have emerged as promising tissue-level biomarkers in this context (9, 10). TLS are ectopic, non-encapsulated lymphoid aggregates that develop in chronically inflamed non-lymphoid tissues, including tumors. In cancer, TLS often contain organized B-cell and T-cell areas, mature dendritic cells, follicular dendritic cells, high endothelial venules and, in mature structures, germinal center-like reactions. These components can support local antigen presentation, lymphocyte recruitment, T–B cell cooperation, B-cell maturation, plasma cell differentiation and humoral immune responses. Thus, TLS differ from diffuse immune infiltration: they represent a higher level of spatially organized antitumor immunity and may indicate whether local adaptive immune responses are coordinated and sustained. Accumulating evidence suggests that TLS are clinically relevant to immunotherapy response (11–13). In melanoma, TLS were associated with improved survival and response to immune checkpoint blockade (14). B cells and TLS have also been linked to enhanced immunotherapy response across several tumor types (15). Importantly, mature TLS were reported to predict immune checkpoint inhibitor efficacy in solid tumors independently of PD-L1 expression and CD8^+^ T-cell density. These findings suggest that TLS may provide complementary information beyond conventional molecular or cellular biomarkers. Rather than simply indicating immune-cell abundance, TLS may reflect the presence of a functional immune niche capable of sustaining local adaptive antitumor responses.

**Figure 1:**
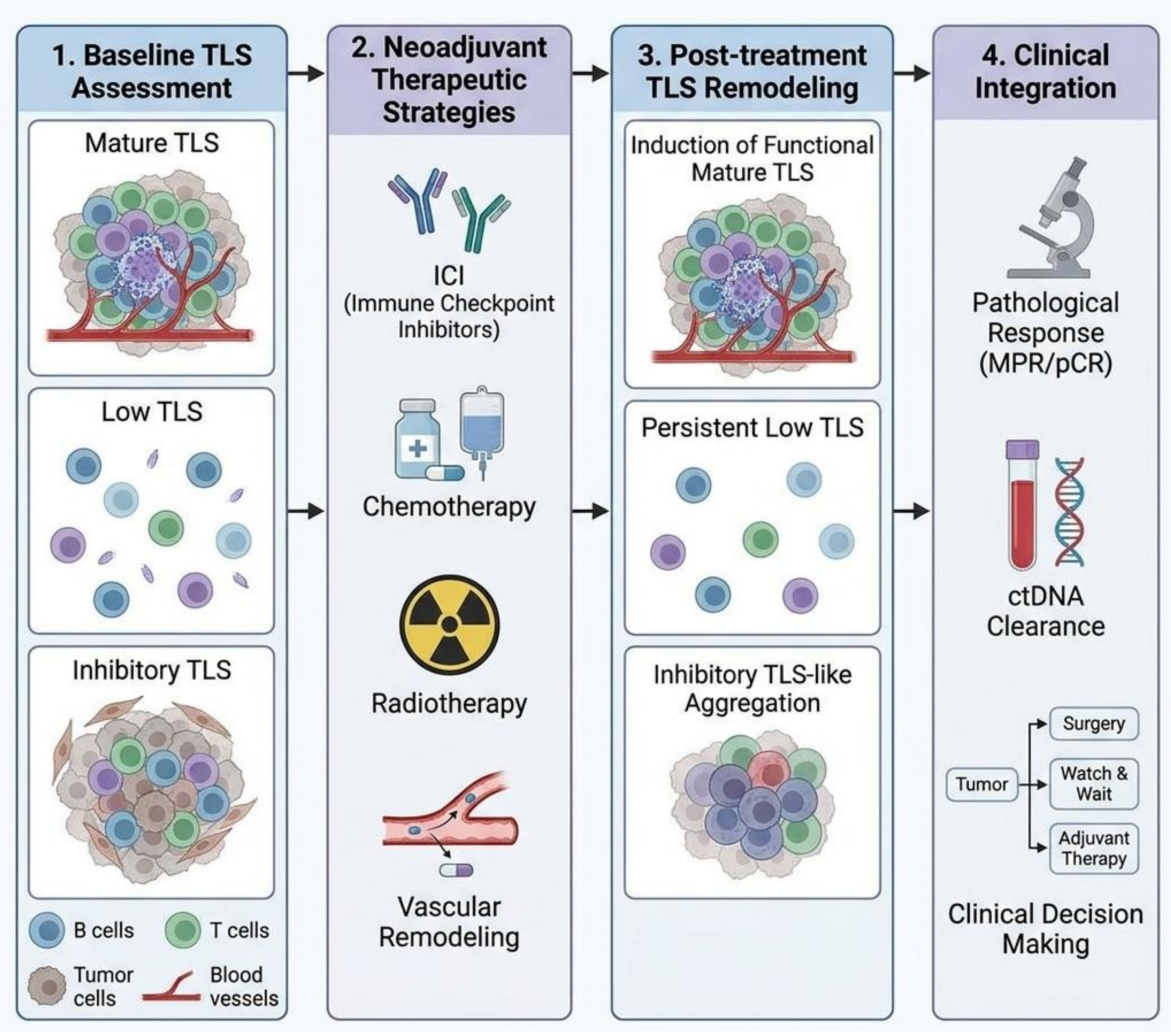
TLS-guided framework for neoadjuvant cancer immunotherapy. TLS may function as both biomarkers and modifiable immune niches in neoadjuvant cancer immunotherapy. Baseline TLS assessment may help stratify tumors into mature TLS-high, TLS-low or immune-cold, and suppressive TLS-like phenotypes. Neoadjuvant therapies may induce or remodel TLS through immune activation, antigen release, lymphocyte recruitment, vascular normalization and stromal reprogramming. Dynamic assessment of post-treatment TLS features, together with pathological response and ctDNA clearance, may support TLS-guided treatment adaptation and future trial design.

The neoadjuvant setting is particularly suitable for studying TLS because paired pre- and post-treatment specimens allow dynamic assessment of tumor immune remodeling (16). In this context, TLS may serve not only as biomarkers of organized antitumor immunity, but also as modifiable immune niches that could be induced or remodeled by rational therapeutic combinations (17). Unlike previous reviews that have mainly summarized the general biology of TLS or their broad association with cancer immunotherapy, this mini-review specifically focuses on the neoadjuvant setting, where baseline biopsies and post-treatment surgical specimens provide a unique opportunity to evaluate TLS remodeling over time. We further emphasize TLS as dynamic tissue-level biomarkers and therapeutically modifiable immune niches that may inform patient stratification, rational combination strategies, response assessment and postoperative risk-adapted management. By integrating TLS biology with neoadjuvant treatment timing, pathological response and clinical translation, this review aims to provide a neoadjuvant-centered framework for TLS-guided cancer immunotherapy. This multi-stage integrated research framework is visually summarized in **Figure 1**.

## 2 Biological basis: TLS as organized immune niches in the tumor microenvironment

### 2.1 Formation and structural composition of TLS

TLS are ectopic lymphoid aggregates that develop in non-lymphoid tissues under persistent inflammatory conditions, including the tumor microenvironment. Unlike diffuse lymphocyte infiltration, TLS display varying degrees of spatial organization and can resemble secondary lymphoid organs in cellular architecture (18). Their development is generally driven by inflammatory cytokines, lymphotoxin-related signaling and chemokine networks that activate local stromal cells and recruit lymphocytes. Key molecular axes include LTα/LTβR, LIGHT, TNF, CXCL13–CXCR5 and CCL19/CCL21–CCR7, which collectively support stromal activation, lymphocyte recruitment and compartmentalization (11, 19).

The roles of TLS should be interpreted according to tumor context. In solid tumors, TLS usually arise within intratumoral or peritumoral regions and are shaped by malignant cells, stromal barriers, tumor-associated vasculature and local inflammatory signals (20, 21). In this setting, TLS may function as spatially organized antitumor immune niches that support local antigen presentation, lymphocyte recruitment, T–B cell cooperation, germinal center-like reactions and humoral antitumor immunity. These features make TLS particularly relevant to tissue-based biomarker assessment, therapeutic remodeling and dynamic monitoring in neoadjuvant immunotherapy. By contrast, in liquid tumors, malignant cells originate from or disseminate within hematopoietic and immune-related compartments, such as bone marrow, peripheral blood, spleen and lymph nodes (22). Therefore, TLS-like immune organization in liquid tumors may overlap with native lymphoid or marrow microenvironments, and its biological interpretation is less directly comparable to TLS formed in solid tumor beds. Because this mini-review focuses on neoadjuvant immunotherapy for solid tumors, the following sections primarily discuss TLS in solid tumor microenvironments, while acknowledging that TLS-like niches in liquid tumors may have distinct biological and clinical implications.

TLS maturation is usually described as a stepwise process. Early lymphoid aggregates consist mainly of mixed lymphocytes and stromal cells without clear T-cell and B-cell segregation. Primary follicle-like TLS show partial organization of T-cell and B-cell areas but lack germinal center activity. Mature TLS are characterized by distinct T-cell and B-cell zones, follicular dendritic cell networks, high endothelial venules, T follicular helper cells and germinal center-like reactions. In lung adenocarcinoma, TLS development has been described as progressing from early CD4^+^ T-cell aggregation to Tfh-cell emergence, B-cell clustering and finally mature structures containing germinal centers, PNAd^+^ HEVs and CD21^+^ FDCs (23).

### 2.2 How TLS support antitumor immunity

The antitumor function of TLS is closely linked to their compartmentalized architecture. First, TLS may provide local sites for antigen presentation, where mature dendritic cells and antigen-presenting B cells interact with T cells and promote local immune activation. Second, HEVs facilitate the entry of circulating lymphocytes into tumor tissues, helping sustain immune-cell recruitment. Third, TLS support coordinated T–B cell interactions, thereby promoting germinal center reactions, class-switch recombination, antibody production and memory responses (24).

These mechanisms suggest that TLS are not passive pathological structures, but active immune niches that can amplify adaptive antitumor immunity within the tumor bed. Functionally, mature TLS may complement draining lymph nodes by supporting local T-cell and B-cell responses. In tumors, TLS-associated B cells can undergo somatic hypermutation and class-switch recombination, and memory B cells and plasma cells have been detected in TLS across several cancer types (25). For neoadjuvant immunotherapy, this is particularly relevant because an organized local immune niche may enhance treatment-induced tumor regression and contribute to postoperative immune surveillance.

### 2.3 Functional heterogeneity of TLS

Although TLS are often associated with favorable antitumor immunity, they should not be considered uniformly beneficial. Their biological and clinical significance depends on maturation status, spatial localization and cellular composition (26). Mature, well-compartmentalized TLS are generally more likely to support productive antitumor immunity, whereas immature aggregates lacking clear organization or germinal center activity may have weaker functional relevance.

TLS may also acquire immunosuppressive features. Enrichment of regulatory T cells, follicular regulatory T cells, regulatory B cells or suppressive macrophages can weaken local antitumor responses. Spatial context is equally important: intratumoral and peritumoral TLS may have different clinical implications, and in some cancers peritumoral TLS have been linked to less favorable immune states (27). For example, spatially distinct TLS can differ in Tfh-cell and Treg-cell enrichment, suggesting that the balance between immune activation and immune suppression shapes TLS function. Therefore, in neoadjuvant immunotherapy, the key question is not simply whether TLS are present, but whether they are mature, functional and oriented toward antitumor immunity.

## 3 TLS as biomarkers in neoadjuvant immunotherapy

### 3.1 Baseline TLS for pretreatment stratification

Baseline TLS may help identify tumors with pre-existing organized antitumor immunity before neoadjuvant treatment (28). Compared with diffuse lymphocyte infiltration, TLS provide spatial information on immune coordination, including B-cell recruitment, T-cell priming, dendritic-cell activation and local humoral responses (29). Therefore, pretreatment TLS may indicate a tumor microenvironment that is already more permissive to immune checkpoint blockade.

However, the predictive value of TLS should not be evaluated simply by their presence or absence. More informative parameters include TLS density, maturation status, intratumoral or peritumoral localization, germinal center-like reactions, HEV density, FDC networks and the balance between effector and suppressive immune cells (29). Mature TLS may provide stronger predictive value than immature lymphoid aggregates because they better reflect functional local adaptive immunity. In this sense, TLS may complement established biomarkers such as PD-L1 expression, TMB and CD8^+^ T-cell infiltration, rather than replace them (30).

### 3.2 Therapy-induced TLS as pharmacodynamic and response-associated biomarkers

The neoadjuvant setting provides a unique opportunity to evaluate TLS dynamically because pretreatment biopsies and post-treatment surgical specimens can be compared within the same patient. In this context, treatment-induced TLS formation or maturation may serve as a pharmacodynamic marker of tumor immune remodeling (31, 32). An increase in mature TLS after therapy may suggest that neoadjuvant treatment has not only reduced tumor burden, but also reorganized the local immune microenvironment toward a more active antitumor state.

This concept is particularly relevant in resectable NSCLC, where neoadjuvant chemoimmunotherapy has been associated with increased CD8^+^ T-cell infiltration, TLS maturation and better pathological response. TLS maturation and abundance have also been linked to survival outcomes, supporting their potential role as tissue-level readouts of effective immune activation (33). Unlike conventional response markers that mainly quantify tumor regression, TLS may provide additional information on whether tumor regression is accompanied by organized adaptive immunity (34).

Nevertheless, the relationship between TLS and pathological regression remains largely associative rather than fully causal. TLS may directly contribute to tumor elimination by supporting local antigen presentation, lymphocyte recruitment and T–B cell cooperation, but they may also emerge as a consequence of effective therapy-induced inflammation. This distinction is important for clinical translation. If TLS are response drivers, therapeutic strategies should aim to induce or mature them; if they are mainly response markers, their value may lie in patient stratification and post-treatment risk assessment. In practice, both roles may coexist depending on tumor type, TLS maturity, treatment regimen and sampling time point.

Therapy-induced TLS should also be interpreted in a treatment-context-dependent manner. Chemotherapy, immune checkpoint blockade, corticosteroids, radiotherapy and local inflammation may have different effects on lymphocyte recruitment, germinal center formation and stromal organization (35). Therefore, post-treatment TLS should be assessed as dynamic immune structures rather than fixed pathological features.

### 3.3 Integrating TLS with long-term prognosis and multimodal biomarkers

Although major pathological response (MPR) and pathological complete response (pCR) are important early endpoints in neoadjuvant trials, long-term outcomes such as event-free survival (EFS), disease-free survival (DFS) and overall survival (OS) remain the ultimate clinical goals. TLS may be relevant to long-term prognosis because they are linked to local immune memory, sustained lymphocyte recruitment and humoral antitumor responses. Mature or intratumoral TLS may therefore indicate not only short-term pathological regression, but also the potential for durable postoperative immune surveillance. However, their prognostic value varies across cancer types and may depend on TLS localization, maturation and suppressive-cell composition (36).

For future neoadjuvant trials, TLS assessment is likely to be most informative when integrated with other biomarkers. PD-L1 expression reflects one axis of immune regulation, TMB estimates antigenic potential, CD8^+^ T-cell density indicates cytotoxic infiltration, and ctDNA reflects tumor burden or molecular residual disease (37). TLS add a distinct layer of information by capturing the spatial organization and functional coordination of local adaptive immunity.

A practical biomarker framework may therefore combine TLS density and maturity with PD-L1, TMB, CD8^+^ T-cell infiltration, IFN-γ or CXCL13-related signatures, TCR/BCR clonality, spatial transcriptomics and ctDNA dynamics (38). For example, mature therapy-induced TLS together with ctDNA clearance may indicate effective tumor eradication and active immune surveillance, whereas TLS-low residual tumors with persistent ctDNA may identify patients who require intensified postoperative treatment. Such integrated models remain to be prospectively validated, but they provide a rational direction for biomarker-driven neoadjuvant trial design (39).

## 4 TLS as therapeutic targets in neoadjuvant immunotherapy

### 4.1 From biomarker to modifiable immune niche

The association between TLS and favorable immunotherapy outcomes raises an important therapeutic question: can TLS be actively induced or remodeled to improve antitumor immunity? This question is particularly relevant in the neoadjuvant setting, where treatment is delivered while the primary tumor, stromal compartment, vascular network and immune infiltrates remain intact. Therefore, neoadjuvant therapy provides a window not only to reduce tumor burden before surgery, but also to reshape the immune architecture of the tumor bed (40).

TLS-directed therapy should not be understood as a strategy to simply increase lymphoid aggregates. The clinically meaningful goal is to promote functional TLS, characterized by coordinated lymphocyte recruitment, HEV formation, mature dendritic-cell activity, T–B cell cooperation, follicular dendritic cell networks, germinal center-like reactions and limited immunosuppressive pressure. This distinction is essential because immature or suppressive TLS-like aggregates may fail to support productive antitumor immunity. Accordingly, therapeutic TLS remodeling should aim to convert poorly organized or immune-excluded tumors into lesions containing mature, spatially coordinated and antitumor-oriented immune niches (41).

To clarify the therapeutic logic of TLS remodeling, TLS-targeted neoadjuvant strategies can be organized into three linked levels: therapeutic inputs, remodeling mechanisms and TLS-related outcomes. Immune checkpoint blockade may amplify pre-existing TLS-associated immunity, whereas chemotherapy, radiotherapy, vaccines and oncolytic viruses may promote antigen release, inflammatory signaling and antigen-directed T- and B-cell priming (42, 43). Innate immune agonists and stromal–vascular remodeling strategies may further enhance dendritic-cell activation, chemokine induction, lymphocyte recruitment and HEV formation. These processes may converge to promote mature TLS with coordinated T–B cell cooperation and germinal center-like reactions. Conversely, incomplete remodeling may leave tumors in a TLS-low state or generate suppressive TLS-like aggregates. Therefore, TLS-directed therapy should prioritize TLS quality, maturity and antitumor orientation rather than simply increasing lymphoid aggregate numbers (44).

### 4.2 Therapeutic priming strategies that may support TLS formation and maturation

Immune checkpoint blockade may enhance the function of pre-existing TLS by relieving inhibitory pathways within organized immune niches. TLS often contain activated T cells, T follicular helper cells, B cells, mature dendritic cells and plasma cells, all of which may be influenced by PD-1/PD-L1 or CTLA-4 blockade. In tumors with mature TLS. In tumors with mature TLS, checkpoint inhibitors may preferentially amplify ongoing local adaptive immunity rather than act on a completely non-inflamed microenvironment. This concept is supported by observations that TLS-rich tumors often show better responses to immune checkpoint blockade (28, 45). In the neoadjuvant setting, TLS have been reported to be enriched in resection samples from responders to nivolumab in NSCLC, and the addition of CTLA-4 blockade to anti-PD-1 plus chemotherapy in operable NSCLC has been associated with increased TLS-related gene expression and improved antitumor activity (46).

However, checkpoint blockade alone may be insufficient in TLS-low or immune-cold tumors, where antigen availability, dendritic-cell activation, lymphocyte recruitment or stromal organization is inadequate. In such tumors, chemoimmunotherapy may provide a broader priming platform by linking tumor-cell killing with antigen release, immunogenic cell death, dendritic-cell activation and inflammatory lymphocyte recruitment (47). In resectable NSCLC, this strategy has provided one of the clearest clinical contexts in which treatment-associated TLS maturation can be evaluated, supporting the concept that cytotoxic therapy and checkpoint blockade may cooperate to generate a more organized antitumor immune microenvironment (48).

Radiotherapy, vaccines and oncolytic viruses represent additional approaches for local immune priming. Radiotherapy may promote antigen release, type I interferon signaling, vascular modulation and chemokine expression, thereby creating a tumor bed more permissive to lymphoid organization (49). Cancer vaccines provide a more antigen-directed strategy; for example, granulocyte–macrophage colony-stimulating factor (GM-CSF)-secreting pancreatic cancer vaccines have been associated with intratumoral lymphoid aggregate formation, and therapeutic vaccination in virus-associated tumors may strengthen antigen-specific T- and B-cell responses (50). Oncolytic viruses may further contribute by inducing tumor-cell lysis, innate immune sensing, inflammatory cytokine production and dendritic-cell activation (51). Although these approaches differ in clinical maturity, they share a common rationale: effective TLS remodeling may require coordinated antigen release, innate immune activation and adaptive immune amplification.

### 4.3 Stromal–vascular remodeling and prevention of non-functional TLS

TLS formation also depends on stromal cells, endothelial cells and chemokine gradients. Fibroblasts and other stromal cells provide structural support and produce chemokines and cytokines such as CXCL13, CCL19, CCL21, IL-7 and BAFF. CXCL13–CXCR5 signaling promotes B-cell recruitment and follicle formation, whereas CCL19/CCL21–CCR7 supports T-cell and dendritic-cell recruitment. LTα/LTβR, LIGHT, TNF, BAFF, APRIL, IL-7 and IL-21 further contribute to stromal activation, HEV development, T–B cell cooperation and follicular organization. These pathways offer potential entry points for inducing lymphoid neogenesis or improving TLS maturation (11, 13, 19).

Preclinical studies support the feasibility of stromal–vascular TLS remodeling. Targeting lymphotoxin or LIGHT to tumors can induce lymphoid-like structures, normalize abnormal vasculature and enhance immunotherapy in resistant tumors (52). STING agonists also represent a promising class of TLS-remodeling agents because they can activate dendritic cells, promote type I interferon-driven inflammation, enhance CD8^+^ T-cell and dendritic-cell infiltration, and support non-classical TLS formation (53). However, these strategies remain less clinically mature than checkpoint blockade or chemoimmunotherapy and should be framed as rational TLS-modulating approaches rather than established neoadjuvant standards.

A major challenge is that TLS induction does not automatically generate beneficial antitumor immunity (54). Some TLS-like structures may remain immature, lacking HEVs, follicular dendritic cell networks or germinal center activity. Others may contain regulatory T cells, follicular regulatory T cells, regulatory B cells or suppressive macrophages, which can weaken local antitumor responses. Therefore, future neoadjuvant strategies should prioritize TLS quality rather than TLS quantity. Functionally favorable TLS should contain organized T-cell and B-cell compartments, mature dendritic cells, HEVs, follicular dendritic cells, Tfh-cell support, plasma-cell differentiation and limited suppressive-cell dominance. Conversely, TLS enriched in Treg/Tfr cells, Breg cells, suppressive myeloid cells or inhibitory checkpoint pathways may require additional modulation through CTLA-4 blockade, myeloid-cell reprogramming, Treg/Tfr control, adenosine-pathway inhibition or stromal normalization.

Overall, TLS-directed therapy should be viewed as a rational extension of tumor microenvironment remodeling. Because TLS formation depends on antigen release, dendritic-cell activation, lymphocyte recruitment, vascular normalization and stromal organization, effective TLS remodeling will probably require rational combinations rather than single-pathway intervention.

## 5 Clinical translation of TLS-guided neoadjuvant strategies

The clinical value of TLS in neoadjuvant immunotherapy should ultimately be judged by whether TLS assessment can inform treatment selection, response monitoring and rational combination design. Rather than treating TLS as descriptive pathological features, an actionable framework should match TLS status with the intervention most likely to remodel the tumor microenvironment. Mature TLS-high or immune-inflamed tumors may be suitable for checkpoint blockade-based regimens; TLS-low or immune-cold tumors may require antigen release, dendritic-cell activation, chemokine induction or stromal–vascular remodeling; and TLS-rich but suppressive tumors may require functional reprogramming of regulatory or myeloid components.

The strongest clinical basis for this framework currently comes from resectable NSCLC. CheckMate 816 established nivolumab plus platinum-doublet chemotherapy as a clinically validated neoadjuvant chemoimmunotherapy platform by improving event-free survival compared with chemotherapy alone (55). The NEOSTAR platform further tested nivolumab plus chemotherapy and nivolumab plus ipilimumab plus chemotherapy in operable NSCLC, providing a setting to examine whether PD-1 and CTLA-4 blockade can reshape local immune architecture beyond cytotoxic tumor killing (56). In parallel, TLS maturation and abundance have been associated with major pathological response and disease-free survival after neoadjuvant chemoimmunotherapy, supporting the use of TLS as a tissue-level immune remodeling readout in this clinical context (57).

Other strategies provide complementary but less mature models for TLS-oriented treatment design. Neoadjuvant durvalumab plus stereotactic body radiotherapy has shown improved pathological tumor killing compared with durvalumab alone in early-stage NSCLC, although TLS-specific endpoints were not prospectively incorporated (58, 59). Vaccine-based approaches provide a more direct lymphoid-aggregate model: in resectable pancreatic ductal adenocarcinoma, neoadjuvant/adjuvant GVAX was feasible, and higher densities of vaccine-induced intratumoral tertiary lymphoid aggregates were associated with longer overall survival (50).

For TLS-low, immune-excluded or stromal-rich tumors, stromal–vascular remodeling and innate immune activation are mechanistically attractive but less clinically mature. LIGHT-based vascular targeting and STING agonist strategies provide preclinical examples of how lymphocyte entry, endothelial activation and lymphoid neogenesis may be promoted, but they should be framed as investigational TLS-modulating approaches rather than established neoadjuvant standards (60, 61).

Taken together, TLS-guided neoadjuvant strategy design should be based on both immune context and evidence maturity. Clinically validated platforms, translational vaccine/radiotherapy models and preclinical TLS-modulating approaches should not be interpreted at the same evidence level. Representative strategies, agents, neoadjuvant contexts, TLS-related mechanisms and translational readiness are summarized in **Table 1**.

**Table 1:** Clinical translation of TLS-guided neoadjuvant therapeutic strategies.

TLS-guided context	Representative strategy or agent	Neoadjuvant or disease setting	TLS-related rationale	Potential clinical use	Major limitation	Translational readiness	Ref
Mature TLS-high/immune-inflamed tumors	Nivolumab + platinum-doublet chemotherapy; nivolumab + ipilimumab + chemotherapy; durvalumab + tremelimumab	Resectable NSCLC; high-risk urothelial carcinoma	Amplifies pre-existing local adaptive immunity within TLS; may enhance T-cell, Tfh-cell, B-cell and DC interactions	Patient stratification	TLS scoring not standardized	Clinically relevant; strongest evidence in NSCLC and urothelial carcinoma	(55, 56, 62)
TLS-low tumors requiring antigen release	Platinum-doublet chemotherapy + nivolumab; atezolizumab- or durvalumab-based chemoimmunotherapy	Resectable NSCLC and other immunotherapy-sensitive tumors	Tumor-cell killing, antigen release, DC activation and TLS maturation	MPR/pCR prediction	Causality unclear	Clinical evidence strongest in NSCLC	(57, 63–65)
Immune-cold tumors requiring local priming	Durvalumab + SBRT; short-course radiotherapy combined with ICI	Localized tumors suitable for preoperative local therapy	Antigen release, type I IFN signaling, chemokine induction and immune-cell recruitment	ctDNA clearance interpretation	TLS endpoints underexplored	Clinically feasible; TLS-specific endpoints remain underexplored	(58, 59)
Antigen-defined or poorly inflamed tumors	GVAX; HPV therapeutic vaccines; engineered oncolytic viral platforms	Pancreatic cancer; virus-associated tumors; accessible lesions	Antigen-directed T/B-cell priming and intratumoral lymphoid aggregate formation	Recurrence risk assessment	Limited clinical validation	Translational evidence; clinical validation needed	(50, 66)
Immune-excluded tumors with vascular/stromal barriers	LIGHT-VTP; CGKRK-LIGHT; anti-VEGF/VEGFR or TGF-β pathway modulation	Tumors with poor lymphocyte entry or dense stroma	HEV induction, vessel normalization, chemokine production and lymphocyte trafficking	Postoperative treatment adaptation	Mostly preclinical	Mainly preclinical or indirect clinical rationale	(60, 67)
TLS-low tumors requiring innate immune activation	ADU-S100/MIW815 and other intratumoral STING agonists; CD40 agonists	Locally injectable or poorly inflamed tumors	Type I IFN response, DC activation, inflammatory chemokines and non-classical TLS neogenesis	TLS induction; ICI sensitization	Delivery and TLS endpoints unclear	Strong mechanistic rationale; clinical TLS evidence limited	(61, 68)
TLS-rich but suppressive tumors	Ipilimumab/tremelimumab-containing regimens; CSF1R inhibitors: pexidartinib; CD73/A2A-axis inhibitors: oleclumab or ciforadenant	Tumors with Treg/Tfr/Breg/TAM-dominant TLS-like structures	Reduces suppressive pressure and may restore TLS-associated effector immunity	TLS maturation as pharmacodynamic readout	Not proven as TLS-directed therapy	Conceptual and translational; should not be framed as proven TLS induction	(69–71)

## 6 Challenges and future directions

### 6.1 Challenges

Despite the growing interest in TLS as biomarkers and therapeutic targets, several challenges must be addressed before they can be incorporated into neoadjuvant immunotherapy decision-making. First, TLS definitions and scoring systems remain inconsistent across studies. Some studies define TLS by lymphoid aggregates on H&E staining, whereas others require B/T-cell compartmentalization, follicular dendritic cell networks, high endothelial venules or germinal center-like reactions. This lack of standardization makes cross-trial comparison difficult. Second, TLS are spatially heterogeneous. Pretreatment biopsies may not accurately represent the whole tumor, while post-treatment specimens may be affected by necrosis, fibrosis and regression-related tissue remodeling. Third, TLS function is context dependent. Mature TLS may support antitumor immunity, but immature or suppressive TLS enriched with Treg cells, Tfr cells, Breg cells or suppressive macrophages may have limited or even unfavorable biological significance (26).

### 6.2 Future outlooks

Future studies should move from simple TLS detection toward functional TLS assessment. An ideal TLS scoring system should integrate density, maturation, spatial localization, HEV/FDC status, T–B cell organization, plasma-cell differentiation and suppressive immune components. In neoadjuvant trials, TLS should be evaluated dynamically in paired baseline and surgical specimens and correlated with pathological response, ctDNA clearance, recurrence risk and long-term survival. More importantly, prospective studies should determine whether TLS-informed stratification can guide treatment adaptation and improve patient outcomes, rather than merely describe immune status. Overall, the future clinical value of TLS will depend on whether they can be transformed from descriptive pathological features into standardized, functional and actionable biomarkers for rational neoadjuvant immunotherapy design.
